# A Virtual Summer Research and Mentorship Program for Underrepresented in Medicine (URiM) Medical Students in Psychiatry

**DOI:** 10.1007/s40596-022-01631-2

**Published:** 2022-04-12

**Authors:** Charles O. Masaki, Lucy Ogbu-Nwobodo, Linda Herrera Santos, Lena B. Faisel, Anisha Lewis, Aldwin Soumare, Nadia Quijije, Rachel A. Ross

**Affiliations:** 1 Harvard Medical School, Boston, MA USA; 2 The University of Toledo College of Medicine and Life Sciences, Toledo, OH USA; 3 University of Connecticut School of Medicine, Farmington, CT USA; 4 Philadelphia College of Osteopathic Medicine, Suwanee, GA USA

To the Editor:

Expanding the health care workforce diversity through mentorship and training is critical in addressing health inequities facing racialized minority populations [1]. These disparities have been further exposed by the COVID-19 pandemic, which has exacerbated long-standing systemic inequities faced by racialized minorities [2].

There is a dearth of physicians from underrepresented in medicine (URiM) backgrounds, defined by the Association of American Medical Colleges (AAMC) as “those racial and ethnic populations that are underrepresented in the medical profession relative to their numbers in the general population” [3]. To address the need for racially diverse clinician-researchers in the biomedical sphere, including in psychiatry, there has been renewed attention to embedding targeted mentorship into research programming, which has been shown to lead to increased advancement of URiM trainees [4]. Here we describe the design and preliminary qualitative outcomes of a virtual summer program offered by Massachusetts General Hospital (MGH)/McLean Hospital. This 8-week program aimed to expose URiM students interested in psychiatry to academic research. It was unique within the institution as it was focused on the topic of psychiatric research. It was structured around three components: mentored research, career mentorship, and community networking. It was funded by a $12,000 administrative supplement offered by the NIH to the R25-funded research training program associated to the MGH/McLean adult psychiatry residency training program.

Three URiM medical students participated in the virtual summer program. The stipend for each was $4000. The students were chosen from a pool of eight applicants based on their interest in psychiatric research and engagement in academic leadership within their communities. A pre-participation survey was administered in which the students identified goals for participation, including to increase research skills, to integrate research into a career in medicine, and to gain long-standing mentorship.

Students were each paired with a research mentor. Mentors, none of whom was from URiM backgrounds due to limited URiM research faculty at our institution, were chosen based on their self-identified interest in participating in the program, track record of engagement in departmental diversity and inclusion efforts, and their lab’s capacity to support virtual research.

Research topics included investigating outcomes in patients treated with transcranial magnetic stimulation, recognizing clinical correlations between multiple variables in an autosomal dominant form of Alzheimer’s disease, and examining brain development in transgender and gender-diverse youth. Students were expected to meet with their lab mentor weekly via video call as well as join lab meetings. The students report that these research experiences fostered skill development that enhanced their curiosity and motivation to continue learning through research. They report:

As a group, we can now work effectively with statistical software, conduct systematic literature reviews, and communicate research findings to the scientific community. We learned to work collaboratively and virtually, and how to manage our time effectively. We identified gaps in psychiatric knowledge and used the research skills acquired to expand our acumen. The program provided us with the confidence and skills necessary to fill in knowledge gaps and thus make contributions that could someday be meaningful to patients*.*

To expand students’ interests and understanding of the career opportunities available for individual training in psychiatry, students participated in department-wide virtual meetings that featured discussions on topics ranging from research communication to inequity in medicine. These meetings were open to all trainees at the institution, and centrally coordinated. An additional weekly mentorship speaker series was developed by the MGH/McLean residency program to expose students to different facets of leadership in psychiatry such as clinical education, medical administration, and academic medicine. Speakers, some of whom identified as URiM, were encouraged to share career obstacles, challenges, strengths, and personal journeys, to allow students to hear varying paths within the field of psychiatry. The students report:

From these mentors we learned strategies of balancing multiple career facets, including life experience, clinical practice, research, and teaching. This affirmed to us that with proper planning and guidance, we too could aim to incorporate various interests into our future careers. Connecting personally with mentors allowed us to learn their unique stories, including the obstacles they overcame to achieve both professional and personal goals, which made our dreams seem much more attainable. Our mentors provided a warm environment that promoted curiosity and inquisitiveness, making us feel included and as integral members of the team.

To enhance the students’ incorporation into the community of psychiatric researchers across our program, the summer students were invited to the MGH/McLean psychiatry residency research program monthly educational dinner curriculum. Each student was paired with a URiM resident peer mentor for additional virtual weekly meetings and monthly virtual social gatherings to expand their peer network. Peer mentors were not directly involved in the student’s core research projects. For the last invited peer network gathering, the participants of the prior year’s in-person URiM program were incorporated to connect across their own experience, and to discuss their trajectory in training. The students report:

Initially, we individually experienced ‘imposter syndrome,’ and were able to discuss these struggles openly with the mentors. Proximity with residents from similar backgrounds, in positions that we aspired towards, helped lessen the ‘imposter syndrome’ by normalizing and demystifying these concerns, and reaffirmed our belief that we deserve to be where we are professionally. Within our student peer group, we discussed our shared experiences as Black medical students, openly admitting when we came across novel concepts and were vulnerable with each other about our knowledge-gaps and experiences. Though we never met in person, we were able to successfully create and foster a close-knit community, allowing for honest reflections on our successes and challenges.

The program culminated in a final presentation during which students presented their research to each other, program faculty, and resident peer mentors. A post-participation survey was administered, with a goal of establishing long-term outcomes as future cohorts participate in the program. The data collected include metrics on career trajectory over time, and quantitation of the impact of the program on the trainees’ career plan. Early data from the survey shows that students successfully participated virtually in their mentors’ labs and attended mentorship and networking events. Two of the three students are continuing to work with their mentors on projects.

The virtual model for the 2020 program was a necessity borne out of the COVID-19 pandemic. This novel format allowed for broader access to some research and mentorship opportunities that may have otherwise been limited by geographical barriers. For instance, it offered more flexibility in arranging meetings, and for increased participation by resident and faculty mentors. It reduced the costs often used for travel and housing, allowing the stipend to be used solely for salary. The success of the virtual model offers an additional tool to consider for the future, even when such a virtual arrangement would not be mandated by a public health crisis.

By intentionally fostering community building between URiM trainees and resident mentors within a research training experience, the program helped to address one of the core barriers postulated to account for the dearth of clinician-scientists from URiM backgrounds, in the form of a lack of role-models from shared underrepresented backgrounds [5]. This program was limited to three students, a number determined by the funding and lab-mentors available to engage virtually with the students. The smaller group may have provided a more comfortable space, one more likely to nurture deeper and more meaningful connections. Though a small group limits the peer network breadth, continuing to engage program alumni from prior years allows for longitudinal network growth, and potential for long-term outcome evaluation.

In summary, we present an innovative model for providing research mentorship to URiM students with limited background in the scientific research methodology. We intend to report on measurable outcomes for future cohorts, but hope this initial letter describing the program and including student reflections provides a framework for scaling similar programs to other institutions.
